# Changes in Maternal Plasma Adiponectin from Late Pregnancy to the Postpartum Period According to the Mode of Delivery: Results from a Prospective Cohort in Rio de Janeiro, Brazil

**DOI:** 10.1371/journal.pone.0158886

**Published:** 2016-07-08

**Authors:** Fernanda Rebelo, Ana Beatriz Franco-Sena, Claudio Jose Struchiner, Gilberto Kac

**Affiliations:** 1 National School of Public Health, Oswaldo Cruz Foundation, Rio de Janeiro, RJ, Brazil; 2 Nutritional Epidemiology Observatory, Department of Social and Applied Nutrition, Institute of Nutrition Josué de Castro, Rio de Janeiro Federal University, Rio de Janeiro, RJ, Brazil; Universite de Montreal, CANADA

## Abstract

**Introduction:**

Maternal plasma adiponectin is inversely related to insulin resistance, atherosclerosis and child health. However, little is known about its concentrations in the perinatal period, especially according to mode of delivery. Our aim is to evaluate the association between mode of delivery and changes in maternal plasma adiponectin from 3^rd^ trimester of pregnancy to 30–45 days postpartum.

**Methods:**

A cohort was recruited in Rio de Janeiro, Brazil, with four waves of follow-up: 5-13^th^, 22-26^th^, 30-36^th^ gestational weeks and 30–45 days postpartum. Eligible subjects should be between 20–40 years of age, be free of chronic and infectious diseases and presenting with a singleton pregnancy. The mode of delivery was classified as vaginal (VD) or cesarean (CS). Plasma adiponectin concentration (μg/mL) was measured using commercial ELISA kits. Statistical analyses included the Wilcoxon rank-sum test and the multiple linear mixed effects model.

**Results:**

A total of 159 participated in the study. Median adiponectin concentrations were higher for the VD group (n = 99; 8.25, IQR: 5.85–11.90) than for the CS group (n = 60; 7.34, IQR: 4.36–9.76; p = 0.040) in the postpartum samples but were not different between the two groups in the 3^rd^ trimester. Women who underwent CS had a lower rate of increase in adiponectin concentration from the 3^rd^ trimester to 30–45 days postpartum compared to those who underwent VD (β = -.15, 95% CI: -.28-.02, p = 0.030).

**Conclusion:**

The CS procedure was associated with lower maternal circulating concentrations of adiponectin at 30–45 days postpartum, compared to the VD.

## Introduction

Adiponectin is a hormone that seems to be inversely related with adverse outcomes throughout life, such as insulin resistance, atherosclerosis, type 2 diabetes mellitus, hypertension, dyslipidemia, metabolic syndrome, hyperuricemia, pulmonary disease and others [1]. During the perinatal period, adiponectin assumes an even more important role, since strong correlations between maternal circulating concentrations of adiponectin and its concentrations in breast milk and the plasma of breastfed infants have been reported [2,3]. Thus, it is plausible that concentrations of this adipokine in maternal plasma may also be related to child health [4,5]. Human milk adiponectin seems to have an important physiologic role in early development and growth of breastfed infants [4].

The trajectory of adiponectin concentrations throughout pregnancy and also in the postpartum period has been explored by several authors [6–12]. However, results from studies assessing the difference between late pregnancy and postpartum concentrations are still contradictory and none of these studies have assessed a possible role of the mode of delivery.

In vitro and in vivo studies have demonstrated decreased synthesis and expression of adiponectin (in blood and adipose tissue) in the presence of systemic inflammation [13,14]. Cesarean sections (CS), as surgeries, are stress factors that may lead to an inflammatory state [15,16] causing changes in women adiponectin concentrations. However, to the best of our knowledge, no study has previously tested this hypothesis.

The occurrence of cesarean section (CS) is alarmingly increasing throughout the world, with frequencies of more than 50% in some countries, including Brazil, the Dominican Republic and Egypt [17]. Systematic literature reviews (SLR) performed in 2006 and 2012 aimed to assess the quality of evidence regarding the risks and benefits of each mode of delivery. The results of these SLR revealed that for the majority of outcomes, such as cardiac arrest, major infections, neonatal mortality and depression, there is insufficient evidence to come to a conclusion; therefore, more research is needed. Moreover, these SLR showed that studies on the association between mode of delivery and hormonal changes are still lacking [18,19].

Considering the increased frequency of CS worldwide, determining all risks and benefits of different modes of delivery is necessary in order to allow women and health professionals to take a conscious decision based on scientific evidences. Therefore, due to the lack of evidence regarding the concentrations of adiponectin in the postpartum period and the potential role of adiponectin in important outcomes of maternal and child health, the present study aims to evaluate the association between mode of delivery and changes in plasma adiponectin concentration from the 3^rd^ gestational trimester to 30–45 days postpartum. Our hypothesis is that women who underwent CS have lower concentrations of adiponectin in the postpartum, compared to those who had a vaginal delivery (VD).

## Methods

A prospective cohort of healthy pregnant women was followed in a low risk prenatal care center in Rio de Janeiro, Brazil, from 2009 to 2012. The study was comprised of four waves of follow-up: 5-13^th^, 22-26^th^, 30-36^th^ weeks of gestation and 30–45 days postpartum. To be eligible for the study, women had to be 20–40 years of age; at 13 weeks of gestation or less; free of chronic non-communicable diseases (excluding obesity) and infectious diseases; and presenting with a singleton pregnancy. Data collection was conducted on previously scheduled days by trained interviewers.

**Fig 1:**
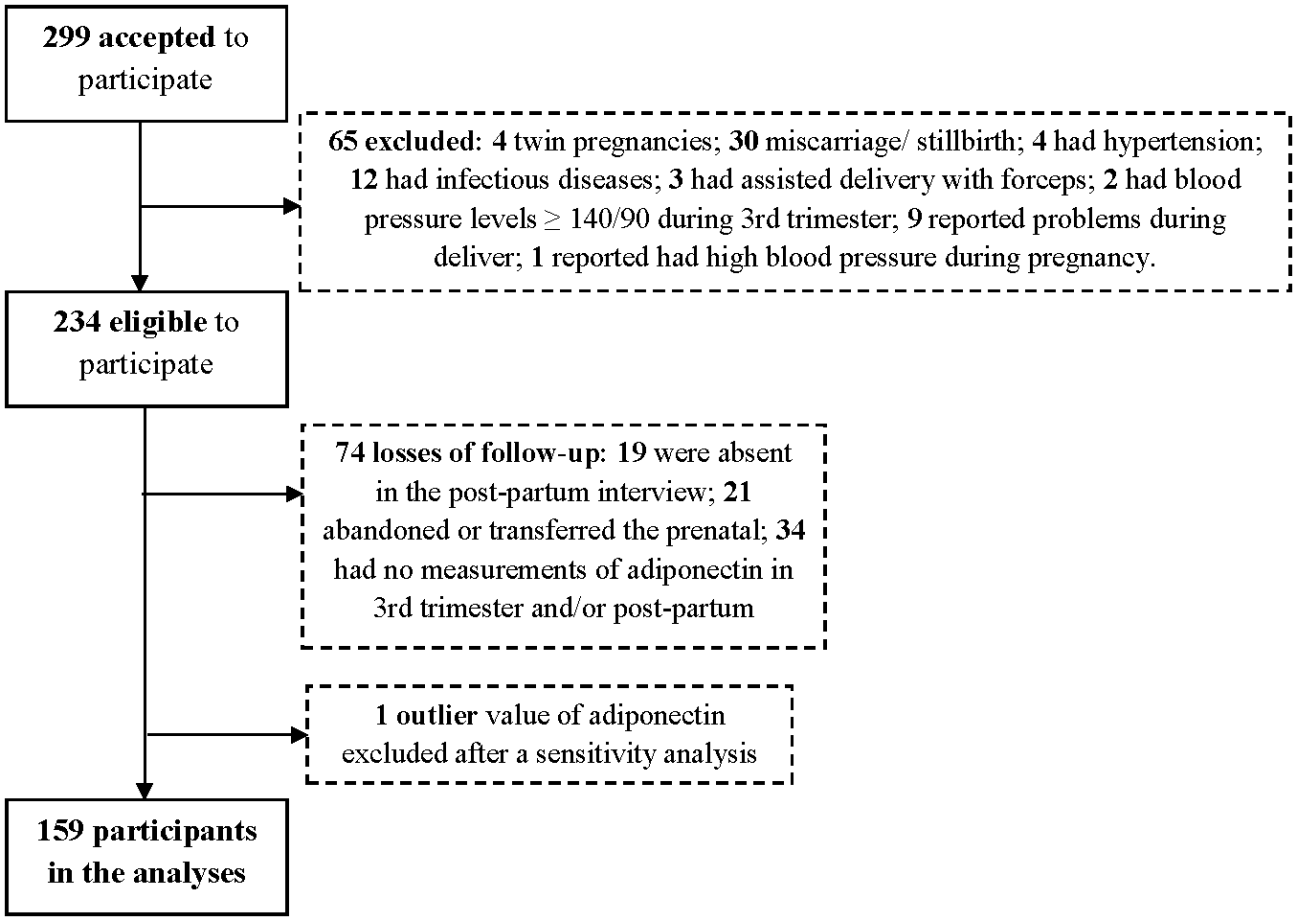
Flow chart illustrating the process of recruitment and follow-up of a pregnancy cohort. Rio de Janeiro, Brazil, 2009–2011.

Fig 1 depicts a flowchart regarding the exclusion criteria and sample size of the study. A total of 299 pregnant women initially accepted to participate in the study. However, we excluded women who presented twin pregnancies (n = 4), those who were diagnosed with an infectious (n = 12) or non-communicable disease (n = 4) after entering the study, those who had a miscarriage or stillbirth (n = 30), those who had blood pressure (BP) higher than 140/90 during the 3^rd^ trimester interview or reported high BP during pregnancy (n = 3), those who reported problems during delivery, such as BP oscillations, tissue damage or excessive bleeding (n = 9), and those whose delivery was assisted with forceps (n = 3). Of the remaining 234 women, 74 were lost to follow-up due to: abandon or transfer to a different prenatal care unit (n = 21), lack of postpartum interview data (n = 19) or lack of adiponectin measurements in the 3^rd^ trimester and/or postpartum period (n = 34). Finally, the adiponectin concentration from one woman was considered an outlier, and this value was excluded after a sensitivity analysis (the removal of the outlier did not change the results). The final sample comprised 159 subjects.

Because the health care center only assisted women with low-risk pregnancies, women with high-risk pregnancies were dropped from the study due to transfer to other centers. We compared the socioeconomic, demographic, anthropometric and reproductive characteristics at baseline of the women in the final sample (n = 159) and of the losses to follow-up (n = 74) to determine whether those who were lost to follow-up left the study randomly (Table 1).

**Table 1:** Comparison between socioeconomic, demographic, anthropometric and reproductive characteristics of women that composed the final sample and losses of follow-up. Rio de Janeiro, 2009–2012.

Variables	Final sample (n = 159)		Losses of follow-up (n = 75)^a^		p-value^b^
	n	%	n	%	
**Age (years)**					
20–29	108	67.9	59	80.8	0.04
30–40	51	32.1	14	19.1	
**Early pregnancy BMI (kg/m**^**2**^**)**					
< 25	92	57.9	44	62.0	0.68
25–30	47	29.6	17	23.9	
≥ 30	20	12.6	10	14.1	
**Education (years)**					
<11	98	61.6	43	61.4	0.98
≥11	61	38.4	27	38.6	
**Skin color**					
White	45	28.3	19	27.5	0.49
Brown	77	48.4	29	42.0	
Black	37	23.3	21	30.4	
**Marital status**					
Lives with a partner	143	89.9	31	88.6	0.81
Does not lives with a partner	16	10.1	4	11.4	
**Parity (number of deliveries)**					
0	52	32.7	35	46.7	0.08
1	60	37.7	26	34.7	
≥2	47	29.6	14	18.7	

Note: BMI = Body Mass Index;

^a^ This n refers to total losses and is not the same for all variables, since some women left the study before we obtained this information.

^b^ p-value refers to chi-square test for proportions.

Blood samples were collected at all waves of follow-up. For the prenatal waves, the women fasted for at least 12 hours prior to blood collection, which occurred between 6:50 and 7:50 am. Postpartum blood was collected without fasting and was usually collected between 10 and 11 am. The samples were centrifuged (5,000 rpm/5 minutes), stored in cylinders containing liquid nitrogen and transported weekly to a -80°C freezer, where they were kept until analysis. Plasma adiponectin concentrations (μg/ml) were measured by enzyme linked immunosorbent assay (ELISA) using commercial kits (Millipore, St. Charles, Mo., USA) with a sensitivity of 0.78 ng/mL. Measurements were made in duplicate and the inter and intra-assay coefficients of variation were 9.9% and 6.7%, respectively.

We also assessed plasma leptin (ng/dL) and insulin (μU/mL) concentrations using ELISA commercial kits (Millipore, St. Charles, MO, USA) with sensitivities of 0.5 ng/mL and 2 μU/mL, respectively. Serum concentrations of C-reactive protein (CRP) were estimated using the immunoturbidimetric method with ultra-sensitive commercial kits (DiaSys Diagnostic Systems GmbH, Germany; sensitivity of 0.05 mg/dL. Serum CRP concentrations were assessed only in the prenatal waves, since in the postpartum only plasma samples were collected.

The mode of delivery was assessed retrospectively in the fourth wave of follow-up through the following structured question: ‘The birth was: normal; forceps; cesarean section; squatting’. The sample had a small frequency of forceps assistance (n = 3). Therefore, we decided to exclude these women since the delivery with forceps assistance is considered VD, but is the clinical alternative for a second stage CS and the outcomes associated with this operative VD are different from those expect for both VD or CS. As no participant had a squatting delivery, the mode of delivery was classified as CS or VD.

Socioeconomic, demographic, reproductive and lifestyle variables were obtained at baseline using structured questionnaires administered by trained interviewers. Information on breast feeding status and birthweight was collected during the postpartum interview. Breast feeding was classified as exclusive (infants received only breast milk and nothing else), mixed (infant received both breast milk and any other food or liquid including water) or none (infant did not receive breast milk).

Maternal body weight (kg) was measured in all four follow-up waves with a digital scale (Filizzola PL 150, FilizzolaLtda, Brazil), and data on maternal height were collected at the baseline of the study using a portable stadiometer attached to the wall (Seca Ltda., Hamburg, Germany). Maternal BMI (weight [kg]/height^2^ [m]) was calculated using the measures of maternal weight throughout the study and the mean height at baseline. Total gestational weight gain (kg) was calculated considering the difference of weights measured in the first and last prenatal visit. The weight change from third trimester to postpartum (kg) was calculated considering the difference of weights measured in the third trimester and postpartum.

Systolic (SBP) and diastolic BP (DBP) were measured using an automated oscillometric BP monitoring system (Omron HEM-742, São Paulo, Brazil). Mean arterial pressure (MAP) was calculated as follows: (SBP + (2*DBP))/3.

Gestational age was calculated based on data from the first ultrasonography examination, if it was performed prior to 26 weeks of gestation. If this measure was not available, the date of the last menstrual period was used to calculate gestational age. The variable ‘time elapsed after conception’ was generated in order to include data regarding postpartum in graphs and longitudinal models. This variable represents the gestational age for the pregnancy follow-up waves and [gestational age at delivery + weeks postpartum at the follow-up visit] for the postpartum period.

### Statistical analysis

A Direct Acyclic Graph (DAG), a type of causal diagram, was constructed using the DAGitty program [20] in order to improve our understanding of the correlation between exposure and outcome and all possible confounding factors. According to this program, the minimal sufficient adjustment set for estimating the direct effect of mode of delivery on plasma adiponectin included birthweight, BP, gestational weight gain, gestational age at delivery and BMI (see S1 Fig, **Supporting Information**).

Means (standard deviation) and proportions of selected variables are presented according to the mode of delivery and were compared using the Student t-test and Chi-square test, respectively. Means and medians (interquartile range) of plasma adiponectin concentrations (1^st^, 2^nd^ and 3^rd^ trimester, postpartum and absolute difference between postpartum and 3^rd^ trimester) were also compared between groups of mode of delivery.

A linear mixed effects (LME) model was constructed to determine if there were any differences in the adiponectin rate of change from the 3^rd^ trimester to 30–45 days postpartum, according to the mode of delivery. LME models capture changes both between and within individuals, accommodate time-dependent and independent covariates, take into account the fact that repeated measures in the same subject are correlated and allow for unbalanced time intervals. The model was adjusted by the variables indicated in the DAG. An additional sensitivity analysis was performed: variables with different distributions between groups of exposure (p < 0.20, according to results of Table 1) were included in the model one by one. However, none of them changed the magnitude of the effect, the direction of the association, or the level of significance. Consequently, they were not kept in the final model. Gestational age was included as both a random and fixed-effect variable. All other variables were treated only as fixed-effect variables.

A graph was constructed (based on the fitted values of the LME model) to illustrate the distribution of plasma adiponectin concentration at each wave of follow-up and longitudinal changes in plasma adiponectin concentration according to the mode of delivery.

A P value < .05 was considered statistically significant. All statistical analyses were performed using Stata Data Analysis and Statistical Software version 12.0 (2011, Stata Corporation).

### Details of ethics approval

The study protocol was approved by the research ethics committee of the National School of Public Health (Protocol number: 33635313.9.0000.5240) and the Municipal Secretary of Health of Rio de Janeiro Municipality (Protocol number: 0139.0.314.000–09). Participants signed informed consent, which was obtained freely and spontaneously after all necessary clarifications had been provided. All ethical procedures of this study related to research involving human beings followed the Brazilian Resolution 466/2012. The participants did not receive any type of compensation.

## Results

The incidence of CS in our study population was 37.74%. The mean values of the characteristics of the participants were as follows: 26.8 (5.58) years of age, 8.43 (3.02) years of education, pre-pregnancy BMI of 24.65 (4.31) kg/m^2^, and gestational weight gain of 11.72 (4.37) kg. Almost one third (32.7%) of the sample was of primiparous women. Women who delivered by CS had significantly higher mean SBP, DBP and MAP compared those who underwent VD (113.58 vs. 111.09; 68.57 vs. 66.47; and 83.57 vs. 81.35 mm/Hg, respectively). All other variables were not different between the CS and VD groups (Table 2).

**Table 2:** Description of sample general characteristics according to the mode of delivery. Rio de Janeiro, Brazil, 2009–2012.

Variables	Total Sample (n = 159)	Vaginal Delivery (n = 99)	Cesarean Section (n = 60)	p-value^a^
	Mean (SD)	Mean (SD)	Mean (SD)	
**Age (years)**	26.80 (5.58)	26.78 (5.64)	26.83 (5.53)	0.52
**Education (years)**	8.43 (3.02)	8.38 (2.89)	8.52 (3.26)	0.40
**Per capita family income (US $)**^b^	287.40 (215.51)	294.08 (227.64)	276.20 (194.82)	0.31
**Parity (number of previous deliveries)**^c^				
0	52 (32.70)	27 (27.27)	25 (41.67)	0.06
≥1	107 (67.30)	72 (72.73)	35 (58.33)	
**Body Mass Index (kg/m**^**2**^**)**^b^				
Pre-pregnancy	24.65 (4.31)	24.32 (4.01)	25.19 (4.76)	0.11
3^rd^ trimester of gestation	28.82 (4.15)	28.40 (3.80)	29.51 (4.62)	0.05
30–45 days postpartum	26.37 (4.11)	26.00 (3.92)	26.99 (4.38)	0.07
**3**^**rd**^ **trimester blood pressure (mmHg)**				
Systolic blood pressure	112.03 (8.90)	111.09 (8.93)	113.58 (8.71)	0.04
Diastolic blood pressure	67.26 (6.68)	66.47 (6.50)	68.57 (6.82)	0.03
Mean arterial pressure	82.19 (6.79)	81.35 (6.70)	83.57 (6.76)	0.02
**Total gestational weight gain (kg)**	11.72 (4.37)	11.69 (4.31)	11.77 (4.52)	0.46
**Weight change from 3**^**rd**^ **trimester to postpartum (kg)**^b^	-8.84 (2.58)	-8.66 (2.52)	-9.14 (2.66)	0.13
**Gestational age at delivery (weeks)**	39.61 (1.43)	39.55 (1.40)	39.69 (1.48)	0.28
**Birthweight (kg)**	3.31 (.46)	3.30 (.39)	3.33 (.57)	0.35
**Breastfeeding (at 30–45 days postpartum visit)**^c^				
Exclusive	105 (66.04)	68 (68.69)	37 (61.67)	0.46
Mixed	47 (29.56)	28 (28.28)	19 (31.67)	
None	7 (4.40)	3 (3.03)	4 (6.67)	
**Serum C-Reactive Protein (mg/L)**	8.37 (6.23)	8.33 (6.25)	8.44 (6.25)	0.46
**Plasma insulin (μU/mL)**	8.75 (8.76)	8.60 (6.94)	8.99 (9.47)	0.39
**Plasma leptin (ng/dL)**	30.86 (21.80)	29.76 (21.91)	32.69 (21.67)	0.21

^a^p-value refers to t-test for comparison of means.

^b^One missing value for the Cesarean Section group.

^c^Values represents n(%) and p-value refers to chi-square test for proportions.

The median postpartum evaluation occurred with 33 days after delivery (IQR: 31.0–37.0). Adiponectin values in the 1^st^, 2^nd^ and 3^rd^ trimesters of pregnancy were not significantly different between the CS and VD groups. Both mean and median values of plasma adiponectin concentration at the postpartum follow-up examination were statistically higher for women who underwent VD compared to those who underwent CS (mean: 9.39 vs. 7.48; median: 8.25 vs. 7.34 μg/mL). The difference between postpartum and 3^rd^ trimester adiponectin concentrations was also significantly higher for the VD group than the CS group (mean: 3.32 vs. 1.21; median: 3.21 vs. 1.74 μg/mL) (Table 3).

**Table 3:** Plasma adiponectin distribution for total sample and according to the mode of delivery categories. Rio de Janeiro, Brazil, 2009–2012.

**Plasma adiponectin (μg/mL)**	**Total sample (n = 159)**	**Vaginal delivery (n = 99)**	**Cesarean Section (n = 60)**	
	**Mean (SD)**			**p-value**^a^
**1**^**st**^ **trimester**^b^	5.56 (2.84)	5.58 (2.85)	5.53 (2.84)	0.45
**2**^**nd**^ **trimester**^c^	6.47 (5.50)	6.81 (6.21)	5.89 (3.97)	0.16
**3**^**rd**^ **trimester**	6.14 (4.05)	6.07 (3.75)	6.27 (4.54)	0.38
**Postpartum**	8.67 (4.97)	9.39 (5.47)	7.48 (3.74)	0.009
**Difference**^d^	2.52 (5.27)	3.32 (5.59)	1.21 (4.43)	0.007
	**Median (IQR)**			**p-value**^e^
**1**^**st**^ **trimester**^b^	4.91 (3.68–6.80)	5.01 (3.68–6.98)	4.80 (3.86–6.69)	0.77
**2**^**nd**^ **trimester**^c^	5.01 (3.49–7.18)	5.08 (3.45–7.40)	4.66 (3.83–6.47)	0.73
**3**^**rd**^ **trimester**	4.98 (3.47–7.34)	5.00 (3.43–7.32)	4.40 (3.60–7.62)	0.94
**Postpartum**	7.75 (5.20–11.25)	8.25 (5.85–11.90)	7.34 (4.36–9.76)	0.04
**Difference**^d^	2.63 (-.21–4.57)	3.21 (-.01–5.97)	1.74 (-.65–3.87)	0.03

Note: SD = Standard deviation; IQR = Interquartile range.

^a^p-value refers to t-test for comparison of means;

^b^Two missing values for the Cesarean Section group.

^c^Two missing values for the Vaginal Delivery group and four for the Cesarean section group.

^d^Refers to the absolute difference between postpartum and third trimester values;

^e^p-value refers to Wilcoxon rank-sum test for comparison of medians.

**Fig 2:**
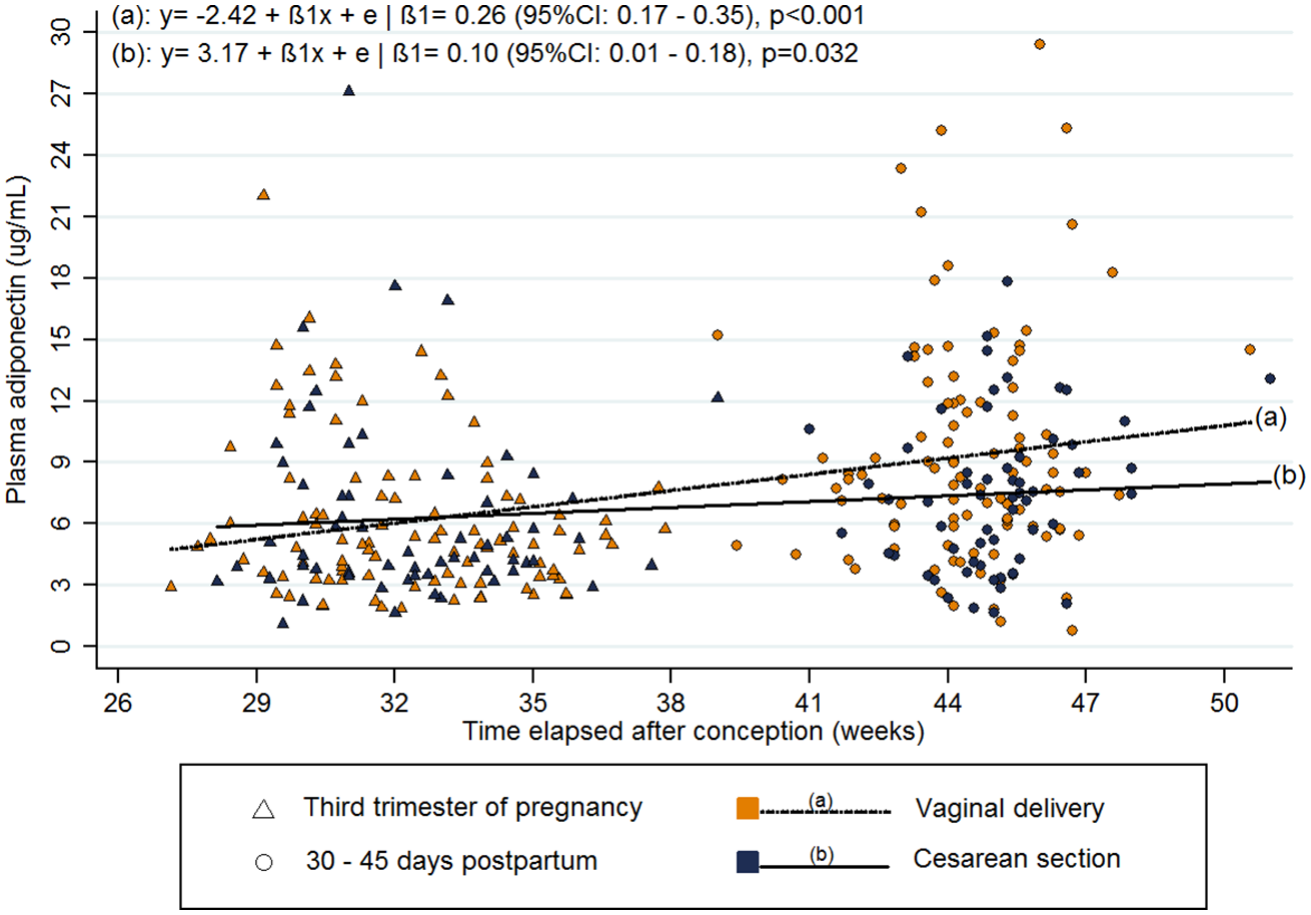
Longitudinal changes in plasma adiponectin from third trimester of pregnancy to postpartum according to the mode of delivery. Rio de Janeiro, 2009–2012.

Women who delivered by CS had a lower rate of increase in plasma adiponectin from the 3^rd^ trimester to the postpartum period than those who underwent VD (β = -.16 [-.30, -.032], p = 0.02). This difference remained even after adjustment for BMI, BP and other confounders (β = -.15 [-.28, -.02], p = 0.03) (Table 4). In both groups, plasma adiponectin concentration increased from the 3^rd^ trimester to the postpartum period. However, the VD group presented a mean rate of increase of .26 μg/mL/week, while the mean rate of increase in the CS group was .10 μg/mL/week (Fig 2).

**Table 4:** Multiple linear mixed effects model for repeated measures of plasma adiponectin at the third trimester of pregnancy and postpartum, according to the mode of delivery and adjusted for confounders. Rio de Janeiro, 2009–2012.

	Plasma adiponectin (μg/mL)			
	Crude		Adjusted	
**Fixed effect**	**β**^a^	**95% CI**	**β**^a^	**95% CI**
Mode of delivery^b^ (Vaginal/ CS)	5.39	.36–10.42	5.10	.02–10.17
Mode of delivery (Vaginal/ CS) ## Time (weeks)^c^	-.16	-.30 –[-.032]	-.15	-.28 –[-.02]
Body Mass Index (kg/m^2^)			-.14	-.28 –[-.002]
Mean arterial blood pressure (mmHg)			.05	-.02–11.48
Gestational weight gain (kg)			.02	-.11 –.15
Gestational age at delivery (weeks)			-.16	-.58 –.26
Birthweight (kg)			.31	-1.04–1.68
Time elapsed after conception (weeks)	.26	.18 –.34	.21	.11 –.30
**Random effect**	**β**^a^	**95% CI**	**β**^a^	**95% CI**
Gestational age (weeks)	.11	.06 –.19	.08	.02 –.29
Intercept	152.91	85.27–274.21	84.93	14.99–481.12
Residual	4.41	1.82–10.67	6.84	2.22–21.10

Note: CS = Cesarean section; n = 159.

^a^β refers to longitudinal linear regression coefficient;

^b^Reference category = Vaginal delivery;

^c^Interaction term between mode of delivery and the time factor, where the β represents plasma adiponectin rate of change over weeks.

## Discussion

Our results showed a different pattern of variation of maternal plasma adiponectin concentrations from the 3^rd^ gestational trimester to the postpartum period according to the mode of delivery. On average, all women had a rise in adiponectin concentrations during this period; however, those who delivered by CS had lower rates of increase of plasma adiponectin concentration than those who underwent VD. Consequently, although no differences were observed between the groups during gestation, women who underwent CS had significantly lower concentrations of this adipokine at 30–45 days postpartum than those who underwent VD.

To our knowledge, this is the first study to evaluate the association between mode of delivery and variations in plasma adiponectin concentration from 3^rd^ trimester of pregnancy to 30–45 days postpartum. The current study had the strength of the availability of repeated measures; thus, we were able to assess adiponectin concentrations before delivery and also observe the rate of change of this hormone over time, from late pregnancy to the postpartum period, to generate more reliable and novel evidence. The proposed objective was carefully assessed, and many confounding factors that may interfere in the estimation of the associations between mode of delivery and adiponectin concentrations over time were considered. A complex causal diagram was constructed, and sensitivity analyses were performed to achieve an unbiased parsimonious model. The effect of mode of delivery on adiponectin concentrations remained invariant after adjustment for confounders, including BMI and BP.

Some limitations need to be noted. First, we did not have data to classify the cases of CS in our cohort as elective or emergency. However, this cohort was recruited from a low-risk prenatal care population (women with high-risk pregnancies were dropped from the study due to transfer to a different prenatal care center) and possible indicators of the requirement of cesarean section (high BP, previous diseases, and twin pregnancy) were exclusion criteria. Furthermore, 3^rd^ trimester CRP levels of women who underwent CS were not significantly different from those who underwent VD, which indicates that the level of inflammation was similar between these two groups at that time. Additionally, the incidence of CS in this cohort is in accordance with a recent investigation of Brazilian women who delivered without complications [21] indicating that our CS cases should be mostly elective.

Secondly, we do not have information on adiponectin isoforms. Some studies suggest that the high molecular weight (HMW) isoform of adiponectin is the most active isoform [22] and that the ratio of HMW to total adiponectin (adiponectin sensitivity index) is a more sensitive marker of the biological activity of adiponectin [23]. Finally, another limitation is the difference in procedures for blood collection between the prenatal and postpartum periods. However, some studies have already observed minor or no diurnal variation in adiponectin concentrations in humans [24,25] and others have documented a lack of interference of fasting on adiponectin concentrations [25,26].

Some authors have already compared postpartum maternal adiponectin concentrations between women who underwent VD and CS, but they did so in other biospecimens and with different time frames. Ozarda et al. (2012)[3] performed a cross-sectional study to evaluate the correlation between adiponectin concentration and inflammation in 157 breast-feeding women that were evaluated between 1 and 180 days postpartum. The authors found no statistically significant differences in serum or milk adiponectin concentrations between the VD and elective CS groups. A possible explanation for these results is that the effect of mode of delivery on adiponectin concentration may differ over time, causing a null effect when it is analyzed over such a long time frame. Additionally, the different postpartum time distribution between the two mode of delivery groups, and the different biospecimens examined (serum and milk, in contrast to plasma in our study) are also sources of heterogeneity.

In the study of Ley et al. (2012)[27], women who underwent scheduled CS had lower levels of breast milk adiponectin at the first postpartum week and the third postpartum month compared to those who underwent VD. The same study found higher concentrations of adiponectin in the first postpartum week in breast milk of women who delivered by unscheduled CS compared to those who underwent VD. The authors did not discuss these contradicting results or clarify what this classification of CS (scheduled or unscheduled) represents.

The rise of adiponectin from late pregnancy to the postpartum period has not been observed consistently by different researchers. The majority of studies have performed this assessment in the early postpartum period (1–14 days after delivery) and observed a tendency of decrease in adiponectin concentrations from pregnancy to that time point [6–9]. In turn, some studies performed this assessment during the late postpartum period (3–12 months after delivery) and reported higher concentrations of adiponectin than during pregnancy, which is consistent with our findings [10–12]. The findings of lower adiponectin concentrations in the early postpartum period and higher adiponectin concentrations in the late postpartum period is logical and is supported by two other previous findings: adiponectin concentrations tend to decrease during pregnancy [7,28] and delivery is an inflammatory process, independently of the mode of delivery [29].

Following this reasoning, we hypothesize that the differences in adiponectin concentration according to the mode of delivery are mediated by the inflammation process. Although both modes of delivery are associated with an inflammatory process, some studies have demonstrated that CS is related to greater inflammation than VD [16,30]. Furthermore, CS can be considered a source of ‘long-term’ inflammation, while VD is a source of ‘short-term’ inflammation. CS inflammation does not end at birth, but rather only after wounds caused by the surgery are completely healed. In an Australian population based survey, 60.7% of women who underwent CS reported pain from wounds at six to seven postpartum months [31]. Unfortunately, we do not have data on inflammatory markers during the postpartum period; therefore, we cannot test this hypothesis. Further studies are needed to elucidate the exact pathway that is responsible for the correlation between mode of delivery and adiponectin concentrations.

The consequences of low adiponectin concentrations, specifically during the postpartum period, are not clear yet. Adiponectin concentration can be considered a marker of cardiovascular risk for the mother and is associated with insulin resistance, obesity and atherosclerosis, as in any other period of life [32]. Because of the strong correlation between maternal plasma adiponectin, breast milk and plasma adiponectin concentrations in breastfed infants [2,3], the effects of maternal plasma adiponectin concentration in children also deserve attention. In an analysis of two independent cohorts, Woo et al. (2009)[5] observed an association of higher breast milk adiponectin concentration with leaner body proportionality over the first six months of life. Other studies have also suggested an inverse association between circulating adiponectin concentration and obesity in children [33,34].

In summary, we could observe an association of CS with lower concentrations of maternal plasma adiponectin at 30–45 days postpartum, compared to women who underwent a VD. These results are of great importance, as they provide new perspectives on the possible outcomes of the mode of delivery. We hope our findings will encourage researchers to study CS-induced hormonal and metabolic changes after delivery. Such studies would improve our understanding of the pathways behind the association of cesarean section and adverse outcomes, which is the first step to developing new therapeutic approaches to avoid these outcomes.

## Supporting Information

S1 Fig Causal diagram for the association between mode of delivery and plasma adiponectin. The minimal sufficient adjustment sets for estimating the direct effect of mode of delivery on plasma adiponectin included: birth weight, blood pressure, gestational weight gain, gestational age at delivery and weight status.
(DOCX)
