# Supplementary material for: Changes in Maternal Plasma Adiponectin from Late Pregnancy to the Postpartum Period According to the Mode of Delivery: Results from a Prospective Cohort in Rio de Janeiro, Brazil
Source: PLoS One. 2016 Jul 8;11(7):e0158886. doi: 10.1371/journal.pone.0158886 (PMC4938429; doi:10.1371/journal.pone.0158886)
Supplement: S1 Fig — The minimal sufficient adjustment sets for estimating the direct effect of mode of delivery on plasma adiponectin included: birth weight, blood pressure, gestational weight gain, gestational age at delivery and weight status. (DOCX) [file pone.0158886.s001.docx]

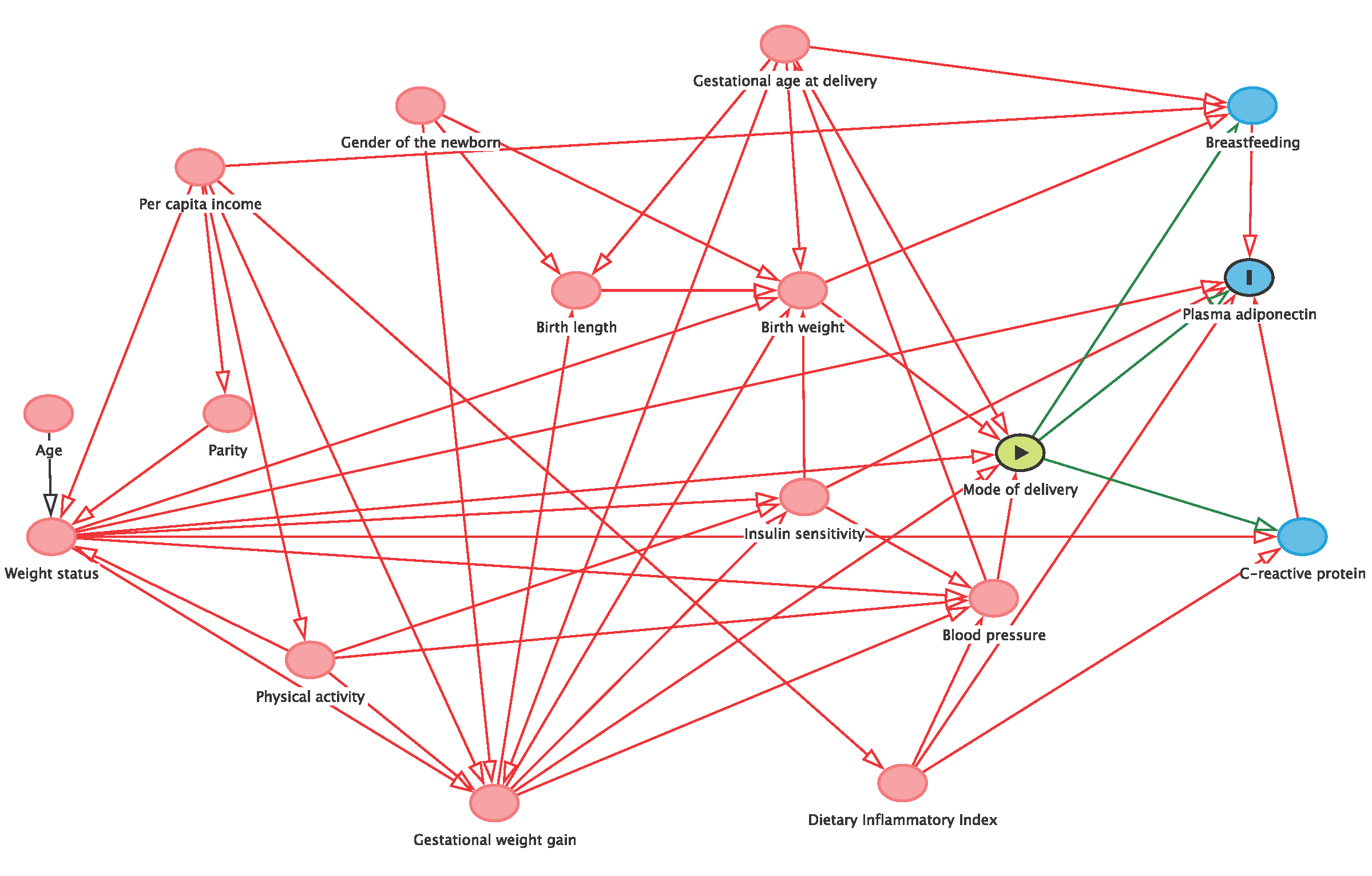


**Figure S1.** Causal diagram for the association between mode of delivery and plasma adiponectin. The minimal sufficient adjustment sets for estimating the direct effect of mode of delivery on plasma adiponectin included: birth weight, blood pressure, gestational weight gain, gestational age at delivery and weight status.
